# Ten-Year Results of Laparoscopic Sleeve Gastrectomy: Retrospective Matched Comparison with Laparoscopic Adjustable Gastric Banding—Is There a Significant Difference in Long Term?

**DOI:** 10.1007/s11695-021-05735-w

**Published:** 2021-10-03

**Authors:** Mario Musella, Giovanna Berardi, Nunzio Velotti, Vincenzo Schiavone, Antonio Vitiello

**Affiliations:** Advanced Biomedical Sciences Department, Naples “Federico II” University, AOU “Federico II”, Via S. Pansini 5, 80131 Naples, Italy

**Keywords:** Sleeve gastrectomy, Laparoscopic adjustable gastric banding, Long-term results, Bariatric surgery

## Abstract

**Background:**

The laparoscopic sleeve gastrectomy (LSG) is the most common bariatric procedure performed worldwide while the laparoscopic adjustable gastric banding (LAGB) has been almost abandoned. Aim of this study was to retrospectively assess 10-year outcomes of LSG through a matched comparison with LAGB.

**Materials and Methods:**

Retrospective search of prospectively maintained database of our university was carried out to find all patients that underwent LSG before December 2010. Each subject with LSG was matched one-to-one with a patient that had undergone LAGB in the same period with correspondent preoperative age, BMI, and sex.

**Results:**

A total of 76 patients underwent LSG before 2010 and were all included in this study; a matched group of 76 out of 178 LAGB patients with 10-year follow-up was retrieved from our database. Comparison between the two groups showed better outcomes after LSG at 1 and 5 years but weight loss was comparable with the LAGB group at 10 years (%TWL 22.2 ± 13 vs 21.2 ± 16.1; *p* = 0.89). No significant difference was found in conversion/removal rate (15.8% vs 18.4%; *p* = 0.67).

**Conclusion:**

LSG is an effective stand-alone bariatric procedure with better outcomes than LAGB in medium term, but results are comparable at 10 years. Subjects undergoing LSG should be informed that conversion to RYGB or OAGB may be necessary to achieve further weight loss or to treat reflux.

**Graphical abstract:**

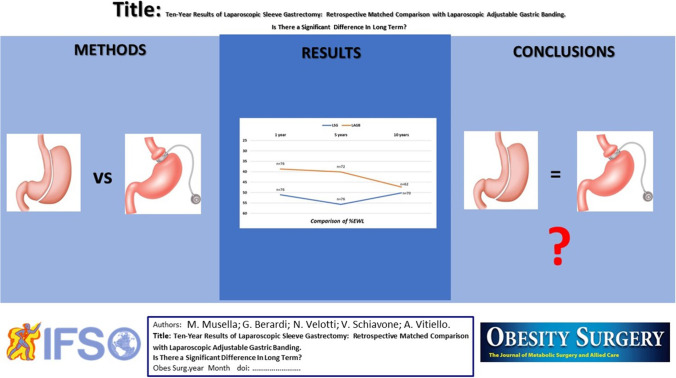

## Introduction

History of restrictive bariatric surgery goes back to the vertical banded gastroplasty (VBG) [1], which was described by Mason in 1982. This intervention was later modified by MacLean et al. [2], who suggested to divide the gastric pouch from the remnant stomach. Aim of this modification was to prevent recanalization between the pouch and the fundus in order to avoid weight regain. Long-term weight loss ranged from 42 to 69%EWL (excess weight loss percent) [3–8], but laparoscopic approach and revisional surgery were challenging tasks after VBG. In the early 2000s, the laparoscopic adjustable gastric band (LAGB) replaced VBG as the most common restrictive procedure. Indeed, LAGB accounted for 24.4% of all weight-loss interventions in 2003 and for 42.3% in 2008 [9, 10]. However, several long-term reports demonstrated a non-response rate up to 40–50% [11–13] and LAGB started to be abandoned.

In 2014, the percentage of bandings performed worldwide decreased to 7.4%, also due to the success of the laparoscopic sleeve gastrectomy (LSG) [14].

Eventually in 2016, LSG overcame the Roux-en-Y gastric bypass (RYGB) as the most common intervention worldwide [15] accounting for 46% of all bariatric interventions in 2018 [16].

However, outcomes of LSG have been recently questioned by several studies, whose results have shown a worrisome rate of postoperative GERD (gastroesophageal reflux disease) [17, 18]. Some articles have also described intestinal metaplasia (Barrett’s disease) after LSG due to the chronic exposure of the lower esophagus to reflux [19, 20].

Since laparoscopic restrictive surgery has been widely performed at our university, some years ago we faced the wave of patients with LAGB coming back to clinic asking for removal or conversion to other interventions. Presently, we are experiencing the same unpleasant situation with subjects who had LSG more than 5 years ago. Therefore, long-term duration of weight loss after the sleeve gastrectomy has been questioned.

Aim of this study was to retrospectively assess 10-year outcomes of LSG through a matched comparison with LAGB with special regard to rates of success (%EWL > 50), non-response (%EWL < 25), weight regain, and conversion.

## Methods

Retrospective search of prospectively maintained database of our university was carried out to find all consecutive patients who underwent LSG at our department before December 2010. Inclusion criteria were age between 18 and 60 years, BMI > 40 kg/m^2^ or > 35 with an obesity-related disease. Subjects with a previous history of bariatric or abdominal surgery were excluded. Each subject who underwent LSG was matched one-to-one with a patient treated with LAGB in the same period with correspondent preoperative BMI, age, and sex (± 1 year age for a given BMI unit).

Collected data at baseline were sex, age, body mass index (BMI), obesity-related diseases, and GERD. Weight loss was analyzed at 1, 5, and 10 years of follow-up. Removal/conversion rate and GERD improvement/worsening were evaluated at 10 years.

Ethics Committee of our institution approved the study and informed consent was obtained from each participant.

### Surgical Technique

Surgical techniques for both procedures have been described in detail elsewhere [21, 22], but a brief description is reported below for completeness of the article.

For LAGB, a total number of 4 trocars (2 × 5 mm; 1 × 10 mm, 1 × 15 mm) were placed. The operation started with the dissection of the gastrophrenic ligament and the opening of the pars flaccida of the small omentum. After the creation of a retrogastric tunnel, the band was drawn along this path and closed. Two gastro-gastric sero-serous nonabsorbable sutures were passed between the gastric fundus and the gastric pouch above the band.

For LSG, a five-trocar approach (3 × 12 mm, 2 × 5 mm) was used. The gastrectomy started 4–6 cm from the pylorus over a 38–40 French bougie. Staple line reinforcements or oversewing is not routinely used at our institution.

### Preoperative Evaluation and Follow-up

All patients were preoperatively evaluated by a multidisciplinary team consisting of endocrinologists, psychiatrists, dieticians, and surgeons. Liquid diet was started on postoperative day (POD) 1 for LAGB and POD 3 for LSG and discharge was planned the day after. Pureed foods were allowed after postoperative day 15 and normal diet after 30 days. Follow-up appointments were routinely planned at 1, 3, 6, and 12 months. After the first year, visits were planned every 6–12 months. Band regulations were decided on the base of symptoms and weight.

### Weight Loss

Weight loss was calculated as percentage of excess weight loss (%EWL), total weight loss percent (%TWL), and excess body mass index loss percent (%EBMIL) using the following formulas:

$$\begin{array}{c}\mathrm{\%EWL}=\left[\left(\mathrm{initial weight}-\mathrm{final weight}\right)/\left(\mathrm{initial weight}-\mathrm{ideal weight}\right)\right]\times 100\\ \mathrm{\%TWL}=\left[\left(\mathrm{initial weight}-\mathrm{final weight}\right)/\left(\mathrm{initial weight}\right)\right]\times 100\\ \mathrm{\%EBMIL}=\left(\mathrm{Initial BMI}-\mathrm{follow}-\mathrm{up BMI}/\mathrm{initial BMI}-25\right)\times 100\end{array}$$

Success at 10 years was defined as %EWL ≥ 50; non-response was set as %EWL < 25 [23], while weight regain was set as %EWL < 50 at 10 years for a patient who had previously achieved %EWL > 50. Percentage of patients with BMI > 35, 35 < BMI < 30, and BMI < 30 kg/m^2^ was also calculated according to Biron’s classification of bariatric results [24].

### Remission from Obesity-Related Disease

Remission of type 2 diabetes (T2DM) was considered as a value of glycated hemoglobin A1c (HbA1C) < 6.5% off antidiabetic medications [25]. Hypertension (HTN) remission was defined as blood pressure < 140/90 off antihypertensive medication [26].

### GERD

At our center, GERD symptoms are routinely investigated in all patients during preoperative and postoperative appointments. According to the Lyon Consensus Conference [27] criteria, de novo GERD was clinically diagnosed in case of new onset heartburn and regurgitation after surgery.

### Complications

Early postoperative complications (bleeding, perforation, staple line leak, stenosis, untreatable vomiting) in the first 30 days and late complications (slippage, erosion/migration, port/tube infection, late staple line leak, and stenosis) were recorded.

### Statistical Analysis

Data are expressed as mean ± SD. Paired *t*-test was used to compare continuous variables as appropriate, while categorical data were compared using the chi-square test and Fisher’s exact test. Significant *p* value was set below 0.05.

## Results

A total of 152 (52 males/100 females) patients were included in this study; 76 patients underwent LSG before 2010 and were all included in this study; and a matched group of 76 out of 178 LAGB patients with 10-year follow-up was retrieved from our database. Baseline demographics are reported in Table 1.

**Table 1:** Baseline characteristics. *BMI*, body mass index; *GERD*, gastroesophageal reflux disease; *LSG*, laparoscopic sleeve gastrectomy; *LAGB*, laparoscopic adjustable gastric band

*Characteristic*	*LSG (n* = *76)*	*LAGB (n* = *76)*	*p value*
*Male gender*	26 (34.2%)	26 (34.2%)	1
*Age (years)*	38.1 ± 9	38.5 ± 11.7	0.38
*Initial BMI (kg/m*^*2*^*)*	45.1 ± 4.8	45.2 ± 4.8	0.16
*Diabetics*	6 (7.9%)	1 (1.3%)	0.11
*Hypertension*	12 (15.8%)	6 (7.9%)	0.13
*Preoperative GERD*	4 (5.2%)	3 (3.9%)	1

### Total Number of LAGB with 10-Year Follow-up

An overall number of 225 patients had undergone LAGB before 2010, but 47 (21%) subjects were loss during follow-up. Therefore, data of 178 (79%) patients are available in our database.

Overall band removal rate at 10 years was 19.1% (*n* = 34); 6 (3.4%) were removed for complications and 28 (15.7%) for insufficient weight loss.

### Follow-up, Complication, and Removal/Conversion Rates

Due to conversion or removal, follow-up at 1, 5, and 10 years was 100%, 100%, and 92.1% in the LSG group and 100%, 94.7%, and 81.6% in the LAGB group.

No major postoperative complication occurred in the two groups.

In the LAGB group, port/tube complications (leak or disconnection leading to infections) occurred in 14/76 (18.4%); drainage, replacement, and repositioning were performed when appropriate [28, 29]. Removal was necessary for 3 (3.9%) subjects after the fifth year for severe dysphagia.

Four LAGBs were removed in the first 5 years due to insufficient weight loss (IWL, 25 < %EWL < 50) or non-response; later, 7 additional patients underwent removal and conversion to LSG (*n* = 4) or OAGB (*n* = 3) for IWL.

In the LSG group, no patient required conversion in the first 5 years, but afterwards 6 patients were converted to one anastomosis gastric bypass/mini-bypass (OAGB) to achieve further weight loss. At the tenth year, 1 subject underwent re-LSG and 3 were submitted to OAGB for weight regain, while other 2 (2.6%) were converted to RYGB for severe reflux.

Rates and causes of removal/conversion are summarized in Table 2.

**Table 2:** Comparison of rates and causes of conversion/removal. *IWL*, insufficient weight loss; *GERD*, gastroesophageal reflux disease; *LSG*, laparoscopic sleeve gastrectomy; *LAGB*, laparoscopic adjustable gastric band

*Reason for conversion/removal*	*LSG (n* = *76)*	*LAGB (n* = *76)*	*p value*
Complications (GERD or band complications)	2 (2.6%)	3 (3.9%)	1
IWL or non-response	10 (13.2%)	11 (14.5%)	1
Conversion/removal rate	12/76 (15.8%)	14/76 (18.4%)	0.67

### Weight Loss

**Fig. 1:**
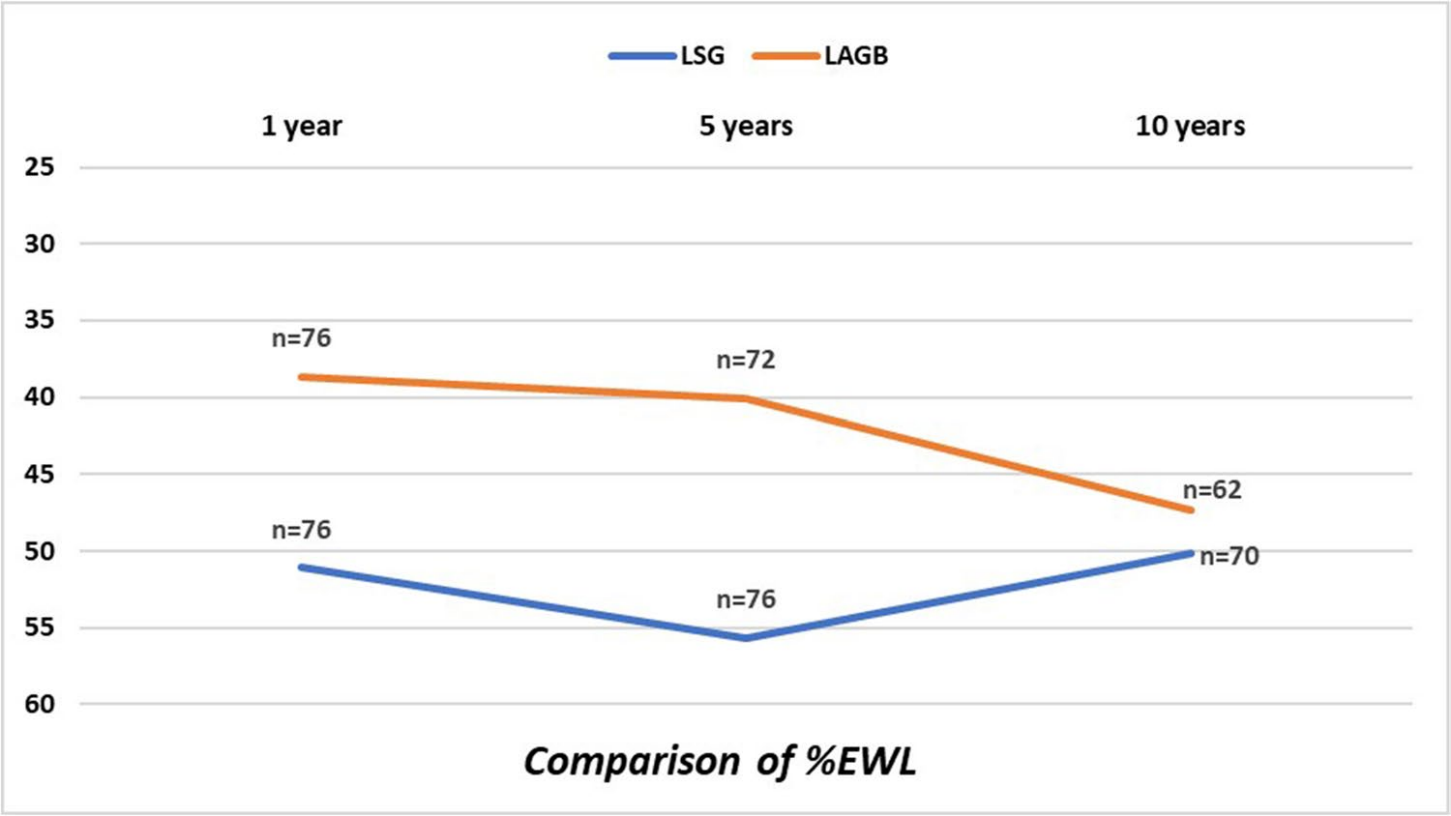
Weight loss comparison between the two groups

Comparison between the two groups showed better outcomes after LSG at 1 and 5 years but weight loss did not result significantly different from the LAGB group at 10 years (Table 3; Fig. 1).

**Table 3:** Comparison of weight loss in the two groups. *BMI*, body mass index; *%EWL*, percentage of excess weight loss; *%EBMIL*, excess body mass index loss percent; *%TWL*, total weight loss percent; *LSG*, laparoscopic sleeve gastrectomy; *LAGB*, laparoscopic adjustable gastric band

	*LSG (n* = *76)*	*LAGB (n* = *76)*	*p value*
BMI 1 year	35.1 ± 5.4	37.7 ± 5.2	< 0.0001
BMI 5 years	33.9 ± 5.3	37.5 ± 6,8	0.0003
BMI 10 years	34.7 ± 5.4	35.6 ± 7.4	0.98
%EWL 1 year	51.1 ± 26.2	38.7 ± 19.1	0.0004
% EWL 5 years	55.7 ± 27.2	40.1 ± 29.3	0.0005
% EWL 10 years	50.1 ± 30.5	47.3 ± 35.2	0.88
%TWL 1 year	22.2 ± 10.7	16.8 ± 8.4	0.0002
%TWL 5 years	30.7 ± 15.5	22.4 ± 17.1	0.0006
%TWL 10 years	22.2 ± 13	21.2 ± 16.1	0.89
%EBMIL 1 year	50.5 ± 26.5	37.4 ± 19.6	0.0002
%EBMIL 5 years	55.1 ± 27.7	38.7 ± 30.1	0.0004
%EBMIL 10 years	49.5 ± 30.9	46 ± 36	0.89
EWL > 50% at 10 years	34/70 (48.6%)	30/62 (48.4%)	0.98
EWL < 25% at 10 years	14/70 (20%)	20/62 (32.3%)	0.11
Weight regain at 10 years	4/70 (5.7%)	10/62 (16.1%)	0.05

**Fig. 2:**
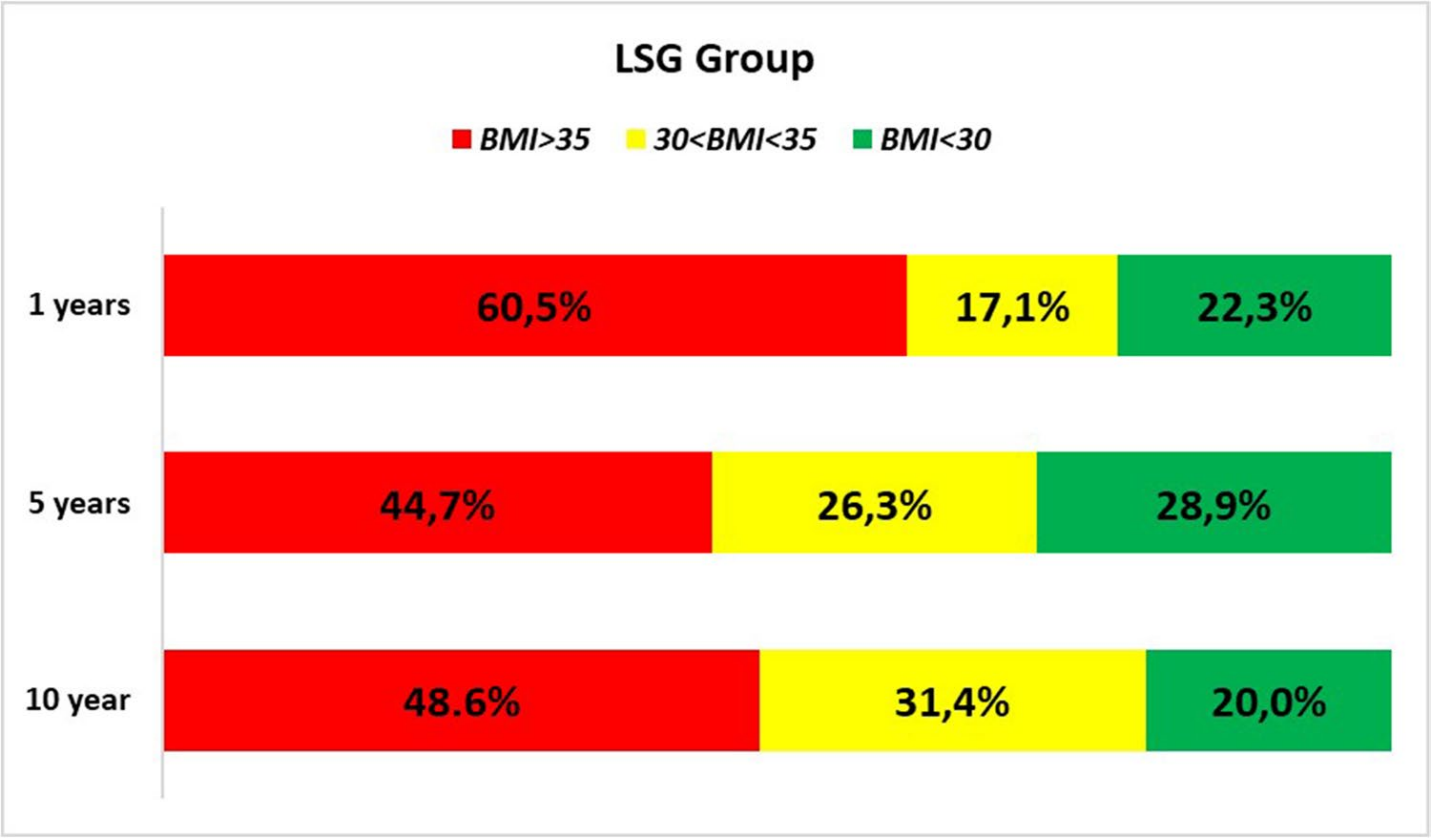
LSG group BMI analysis

**Fig. 3:**
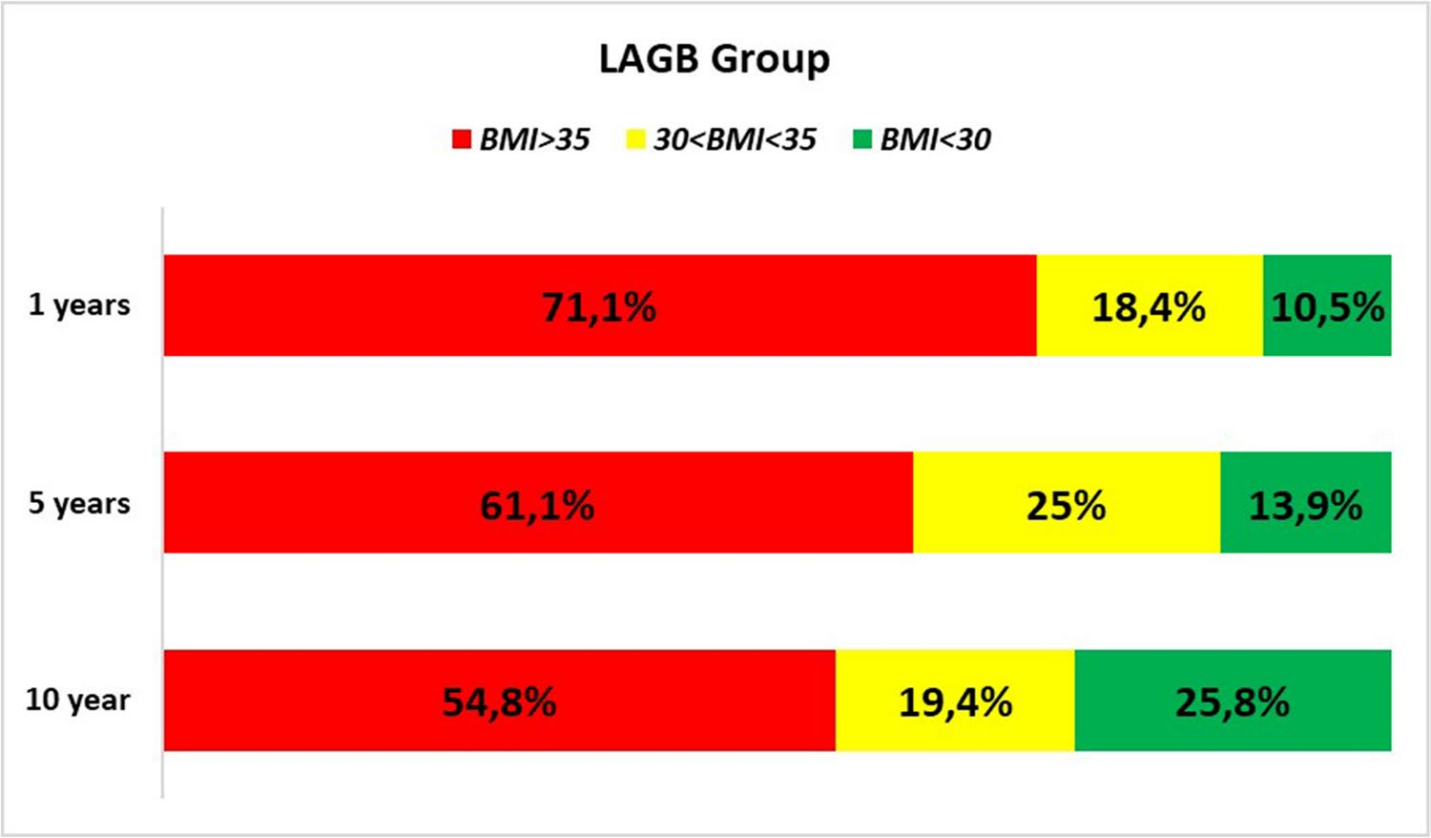
LAGB group BMI analysis

Analysis of BMI according to Biron’s classification showed that half of patients in both groups had BMI > 35 at 10 years (Fig. 2 and 3).

### Remission from Obesity-Related Disease and GERD

After 10 years, all patients with preoperative diabetes were still taking drugs; rate of remission from hypertension was 58% after LSG and 16.7% after LAGB.

Eighteen cases of de novo GERD occurred after LSG and 4 after LAGB; symptoms did not improve for the only patient who suffered from preoperative reflux (Table 4).

**Table 4:** Remission from comorbidities and GERD (gastroesophageal reflux disease). *LSG*, laparoscopic sleeve gastrectomy; *LAGB*, laparoscopic adjustable gastric band

	*LSG*	*LAGB*	*p value*
Remission from hypertension	7/12 (51.4%)	1/6 (16.7%)	0.15
Remission from diabetes	0/6 (0%)	0/1 (0%)	1
Remission from GERD	0/4 (0%)	0/3 (0%)	1
De novo GERD	18/70 (25.7%)	4/62 (6.5%)	0.004

## Discussion

Currently VBG is not included in the guidelines of bariatric surgery while LSG is the most common intervention performed worldwide and LAGB has been almost abandoned.

Main reason of the decline of LAGB was the rate of non-response or weight regain, as reported in series with medium [30] and long-term follow-up [31, 32]. A systematic review of studies with 10-year follow-up and a total of 9706 patients [33] showed an average %EWL of 49 at 10 years with a 30% removal rate. Nevertheless, recent studies reported that, considering its reversibility and safety, LAGB could still be proposed for selected patients [34].

Conversely, LSG was initially introduced by Marceau et al. [35] as first part of the duodenal switch operation in order to preserve vagal innervation and pyloric function. Subsequently, Regan et al. [36] proposed a staged procedure also for gastric bypass, mostly to overcome the challenge of laparoscopic surgery in patients with BMI > 60 kg/m^2^. Since postoperative outcomes demonstrated low morbidity and satisfactory weight loss, LSG achieved the status of a bariatric intervention [37].

Short-term studies (1–3 years) on LSG have reported an excellent outcome in terms of excess weight loss (%EWL), which is comparable to values after RYGB [38]. Mid-term reports (5–7 years) have shown less successful results, since weight recidivism rate was estimated to be 27.8% with a range of 14 to 37% [39–41]; the SM-BOSS [42] study showed that BMI loss peaked at 2 years after SG (74.7%) but decreased by the end of the fifth year to 61.1%. In a previous retrospective comparison [40], patients with LSG achieved better weight loss than LAGB at 5 years, but comparable remission from comorbidities. Medium-term studies also demonstrated a rate of remission from diabetes and hypertension of almost 70% [43] after LSG.

Surprisingly, despite LSG has been widely performed in the last 20 years, there is a lack of long-term studies (8–10 years). Felsenreich et al. [44] reported 54% EWL after a mean follow-up of 10.8 years with sample size of 53 patients, while Arman et al. [45] found 62.5%EWL in 110 patients.

Very recently an article with results beyond 10 years has been published: even if LSG provided a long-term %EBMIL ranging from 51 to 54%, high incidence of insufficient weight loss and de novo reflux was observed; conversion to other interventions was necessary in 19.2% of cases.

Even if some studies reported improvement of symptoms after SG [46, 47], most articles show a worsening of preoperative reflux and de novo GERD [48]. Undoubtedly, the main reason for new onset heartburn is the increased intragastric pressure (IGP) caused by a sleeved stomach, resulting in a decreased compliance. Also, technical mistakes could lead to a stenotic or twisted sleeve, which causes regurgitation of acid content into the esophagus.

Braghetto and Csendes [49] first reported an incidence of 1.2% of BE at 1 year after LSG; lately other authors reported a rate of 15–17% after a longer follow-up (5 to 10 years) [20]. A recent systematic review showed a rate of de-novo GERD of 20% [50] after LSG, while a meta-analysis found that the increase of postoperative GERD was 19% and de novo reflux occurred in 23% [51] of patients. Our study shows an important incidence of clinical GERD after LSG, being one fourth of patients diagnosed with heartburn and regurgitation at 10 years.

Even if the outcomes of the present study match the results of the abovementioned literature, comparison between LAGB and LSG showed interesting results at 10 years.

In short and medium term, superiority of LSG is clear, but the two procedures show a similar outcome at 10 years. Success of LSG is mainly due to the massive results achievable in the first postoperative years, when weight loss is definitively better and probably comparable to RYGB. However, as said before, after the fifth year a progressive weight regain begins which may be irreversible in the long term.

Remarkably, the curve of weight loss after LAGB continuously slopes down, while the curve after LSG starts to rise after the fifth year. Explanation of this different trend probably relies in the different history of the two interventions. In the last 10 years, LAGB has been considered not very successful, then patients with initial signs of IWL or non-response have rapidly undergone removal or conversion. Subsequently, there is an early drop-out of subjects with bad results from the LAGB group; therefore, only patients with better results are included in the long-term follow-up.

On the other hand, weight regain was not expected after LSG and conversion to another procedure was often delayed. Indeed, half of conversions after the sleeve occurred at the tenth year. Probably, the risk of regaining kilos after LSG is higher for those subjects with IWL. Indeed, we found that only 5.7% of patients with %EWL > 50 before year 5 failed to maintain this result in long term. In this view, it is interesting that conversion/removal rate was comparable at 10 years and that the main reason for revisional surgery was IWL or non-response in both groups.

It is undeniable that all surgical units are recently facing a wave of patients with LSG requiring conversion, as happened years ago with subjects who had undergone LAGB. This trend will probably continue in the next years, since 48.6% of sleeved subjects still have indication for bariatric surgery (BMI > 35 kg/m^2^ in long term).

Nevertheless, it must be underlined that almost half of LSG patients have conserved a successful mean weight loss (EWL > 50%) at 10 years, proving that the sleeve gastrectomy deserves to be considered a stand-alone procedure.

In our bariatric center, patients with morbid obesity and multiple comorbidities are usually submitted to malabsorptive procedures; therefore, numbers are too small to draw serious conclusions on comorbidities remission, but improvement really happened only after LSG.

### Strength and Limitations

The present study reports outcomes of a matched retrospective study with acceptable sample size considering the long-term follow-up.

However, comparison could be biased by initial technique for LSG, which was fashioned less tightly over larger bougies. Currently, the sleeve operation is performed over a 38 French tube starting 4 cm from the pylorus; some authors [52] have stated that a radical antrectomy could improve and accelerate weight loss.

Also, GERD assessment was only clinical, and although international validated standard questionnaires were used, and data from instrumental investigations were often unavailable.

## Conclusion

LSG is an effective bariatric procedure in short and medium term with clear superiority over LAGB. However, after 10 years, weight loss and conversion rate were comparable between the two procedures and 15.8% of LSG patients required reoperation. Long-term RCT are needed to better understand the rate for conversion.

Patients with morbid obesity should be adequately counseled to LAGB or LSG. Those subjects unwilling to undergo an irreversible, even if more effective, procedure could be submitted to LAGB; patients selected for LSG should be informed that conversion to RYGB [53] or OAGB [54] may be necessary to achieve further weight loss or to treat reflux.
